# Dynamic Changes in Microbiome and Metabolome during Sun-Drying of Oysters (*Crassostrea gigas*), a Traditional Procedure in South China

**DOI:** 10.4014/jmb.2312.12033

**Published:** 2024-07-17

**Authors:** Jiannan Liu, Mingjia Yu, Xiaobo Wang, Ming Qi, Minfu Wu

**Affiliations:** Department of Food Science, Foshan Polytechnic, Foshan 528137, P.R. China

**Keywords:** *Crassostrea gigas*, sun-drying, microbiota, metabolites, pathogens, nutrition

## Abstract

Sun-drying constitutes a traditional method employed in the preparation of dried oysters within the coastal regions of South China. However, its ramifications on nutritional attributes and the genesis of flavor-contributory compounds in the resultant dried oysters remain significantly unexplored. This research endeavors to scrutinize the repercussions of the production process on the microbiota and metabolites within dried oysters. Utilizing 16s rRNA amplicon sequencing, the identification of 409 operational taxonomic units (OTUs) ensued, wherein *Proteobacteria*, *Bacteroidetes*, *Firmicutes*, *Tenericutes*, and *Actinobacteria* surfaced as the primary pathogenic bacteria present in oyster samples. Analysis of the dried oyster sample metabolomes via LC-MS unveiled a discernible augmentation in compounds associated with steroid hormone biosynthesis, arachidonic acid metabolism, biosynthesis of unsaturated fatty acids, and linoleic acid metabolism throughout the entirety of the drying process. Subsequent exploration into the association between metabolites and bacterial communities highlighted the prevailing coexistence of *Mycoplasma*, *Psychrilyobacter*, and *Vibrio* demonstrating negative correlations with a substantial number of metabolites across categories including organic acid and its derivatives, nucleotide and its metabolites, free fatty acids, and amino acids. Conversely, *Shewanella* and *Arcobacter* exhibited positive correlations with these metabolite categories. This exhaustive investigation offers invaluable insights into the dynamic alterations within the microbiota and metabolites of dried oysters across diverse drying periods. These findings are anticipated to significantly contribute to the advancement of production techniques and the formulation of enhanced safety measures for the processing of dried oysters.

## Introduction

*Crassostrea gigas* is one of the widely farmed seashell species in China, and one of the world's popular aquatic foods [1, 2]. They are considered a nutrient-rich source of protein, unsaturated fatty acids, vitamins and minerals [3, 4]. The predominant oyster species retailed in the coastal regions of southern China is the Pacific oyster [5]. The drying process is usually the most common means of storing oysters. Various drying techniques, such as vacuum freeze-drying, heat-pump-drying, hot-air drying, and sun-drying, are employed in the dehydration process of oyster [6, 7]. Divergent drying methods may impart distinct flavor profiles to the resultant dried oyster. In the context of China, sun-drying stands out as the prevailing and cost-effective approach among these methods [8-10], and the dried oyster was also called “Haochi” in South China and usually eaten by people at traditional festivals.

Raw oysters of the *C. gigas* species constitute a multifaceted ecosystem characterized by a diverse assemblage of active microorganisms. Ensuring the safety of oyster products is imperative for safeguarding consumer health [11]. The prevalent bacteria identified in deteriorated Pacific oysters cultivated through conventional methods were *Pseudomonas* and *Vibrio* [3, 12]. *V. vulnificus* and *V. parahaemolyticus* constitute the predominant life-threatening foodborne pathogens and have been documented in instances of *Vibrio*-associated wound infections [11]. *Vibrio* infections associated with eating uncooked or undercooked oysters continue to increase [13]. Especially, *V. vulnificus* is responsible for 95% of seafood-related deaths, presenting a major food safety concern [14].

The presence of the main nutritional and flavor components of dried oyster, including steroid hormone, arachidonic acid and unsaturated fatty acids [15, 16]. And the impact of retained microorganisms on various components remains uncertain. In order to elucidate potential detrimental alterations in the microbial community, identify the key microbial species retained during the oyster drying process, and examine their associations with the nutritional and flavor components of dried oyster, we conducted a comprehensive analysis of microbiota and metabolite changes across four distinct drying periods. The findings from this investigation offer valuable insights applicable to the seafood industry.

## Materials and Methods

### Preparation of Dried Oysters

Freshly purchased shell-on oysters were cleaned after shucking and subsequently placed under direct sunlight, with daily flipping of the oysters at 10 AM. Samples were collected at four time points: before sun exposure (OGT0), 1 d after sun exposure (OGT1), 2 d after sun exposure (OGT2), and 4 d after sun exposure (OGT4). Four biological replicates (oyster samples) were collected at each timepoint. The samples were then divided into two portions for subsequent processing. One part was homogenized for total viable bacterial count and pH measurement, while the other part was rapidly frozen in liquid nitrogen and stored at -80°C for subsequent 16s rRNA sequencing and metabolome analysis.

### Determination of Total Viable Count (TVC) and Moisture

Approximately 0.25–0.5 g of oyster samples were placed in sterile homogenization bags containing 90 ml sterile physiological saline. The samples were homogenized for 40 s to obtain a 1:10 dilution. One milliliter of the diluted sample was aseptically transferred to 9 ml of sterile physiological saline to prepare a 1:100 dilution. This process was repeated to create a series of tenfold dilutions. Plate spreading on PCA agar medium (Sangon Biotech, China) was performed with an aliquot of 100 μl from each dilution, and the plates were incubated at 36°C ± 1 for 48 h before counting. Total viable count was measured according to the Chinese GB standards (GB4789.2-2010)(National Food Safety Standard Food microbiological examination: Aerobic plate count. Chinese Standards Press; China: 2010). The moisture content of samples during the sun-drying process was measured based on GB 5009.3-2016 method (National standard for food safety-Determination of moisture in foods. China Food and Drug Administration; Beijing, China: 2016).

### DNA Extraction and 16S rRNA Sequencing

One milliliter of the 1:10 sample dilution after homogenization was used for genomic DNA extraction using the FastDNA Spin kit for soil (MP Biomedicals, USA) according to the manufacturer’s instructions. The V3–V4 region of the 16S rRNA gene was amplified using specific primers (341F: CCTACGGGNGGCWGCAG and 806R: GGACTACHVGGGTATCTAAT) carrying barcodes [17]. Gel purification was performed to recover the PCR amplification products, which were then quantified using a QuantiFluorTM fluorometer. In the OGT1 and OGT4 groups, one sample from each group was excluded from sequencing because of the inadequate quality of the 16S rRNA sequencing library. Purified amplification products were equally pooled, ligated with sequencing adapters, and used to construct sequencing libraries. High-throughput sequencing was performed on an Illumina MiSeq PE300 platform (Illumina, USA) according to the standard protocols of Majorbio Bio-Pharm Technology Co., Ltd., China).

### Bioinformatics Analysis

Low-quality reads were subjected to quality filtering using FASTP v0.18.0 [18]. Subsequently, the paired-end reads were assembled into tags using the FALSH tool v1.2.11, and then filtered to obtain the clean tags [19, 20]. The clean tags were subjected to operational taxonomic unit (OTU) clustering using the UPARSE algorithm within USEARCH software v9.2.64 [21, 22]. In addition, the UCHIME algorithm was employed to identify and remove chimeric tags [23]. OTU abundance profiling was performed based on effective tags.

The Naïve Bayesian assignment algorithm implemented in RDP Classifier v2.2 was utilized for species annotation based on the SILVA database v132 [24]. Alpha diversity analysis, principal coordinate analysis (PCoA), and anosim analysis were conducted using QIIME v1.9.1 and Vegan package v2.5.3, respectively [25, 26]. Indicator species analysis was performed using the Labdsv package v2.0-1 [27].

### Metabolite Extraction, Detection, and Analysis for Untargeted Metabolomics

The samples were homogenized in a liquid nitrogen environment, and approximately 20 mg (±1 mg) of each sample was added to 400 μl of 70% methanol aqueous internal standard extraction solution. The mixture was subjected to oscillation at 1,500 rpm for 5 min, followed by a 15-min incubation on ice. Afterward, the supernatant was collected through two consecutive centrifugation steps at 12,000 ×*g* (4°C) for 10 min and 3 min, which was used for liquid chromatography-tandem mass spectrometry (LC-MS/MS) analysis, utilizing an LC-30A ultra-high-performance liquid chromatography system (LC-30A, Shimadzu, Japan) coupled with a TripleTOF 6600+ mass spectrometer (SCIEX, USA).

For LC-MS/MS analysis, 2 μl of the supernatant was injected into a C18 chromatography column (Waters ACQUITY UPLC BEH C18 1.8 μm, 2.1 mm × 100 mm) at a flow rate of 0.40 ml/min. The mobile phases consisted of ultra-pure water (0.1% formic acid) as phase A and acetonitrile (0.1% formic acid) as phase B, with the column temperature maintained at 40°C. LC-MS/MS data were acquired using the TripleTOF 6600+ system in information-dependent analysis mode, with an ion spray voltage floating of 5000 V in positive mode or -4000 V in negative mode, respectively.

The original LC-MS/MS data were converted into the mzXML format using ProteoWizard [28]. Peak extraction, alignment, and correction were performed using the XCMS program and SVR method [29]. Peaks with a missing rate greater than 50% in each group of samples were filtered out. Metabolite identification was performed by searching against a custom database that integrates public and AI prediction databases using the metDNA method. Orthogonal partial least-squares discriminant analysis (OPLS-DA) analysis was conducted using MetaboAnalystR v1.0.1 [30, 31], with the threshold for identifying inter-group differentially accumulated metabolites (DAM) set at VIP > 1 and FDR-adjusted *P* < 0.05 (Student’s *t*-test). The Short Time-series Expression Miner (STEM) tool was used to analyze temporal metabolomic patterns. Significant profiles of metabolites were selected with an FDR-adjusted threshold of *P* < 0.05. Metabolite annotation was carried out using the KEGG Compound Database (http://www.kegg.jp/kegg/compound/) [32], and pathway enrichment analysis was performed based on hypergeometric testing.

### Statistical Analysis and Visualization

Spearman’s correlation between microbiota and metabolites was computed using the Hmisc package. A threshold of a correlation coefficient greater than 0.7 and an FDR-adjusted *P* less than 0.05 were applied for filtering. A bacterial metabolite network was constructed based on the correlation data, and a correlation network graph was visualized using Cytoscape is 3.10.1[33].

## Results and Discussion

### Variation in TVC and pH of Oysters during Preparation

**Fig. 1:**
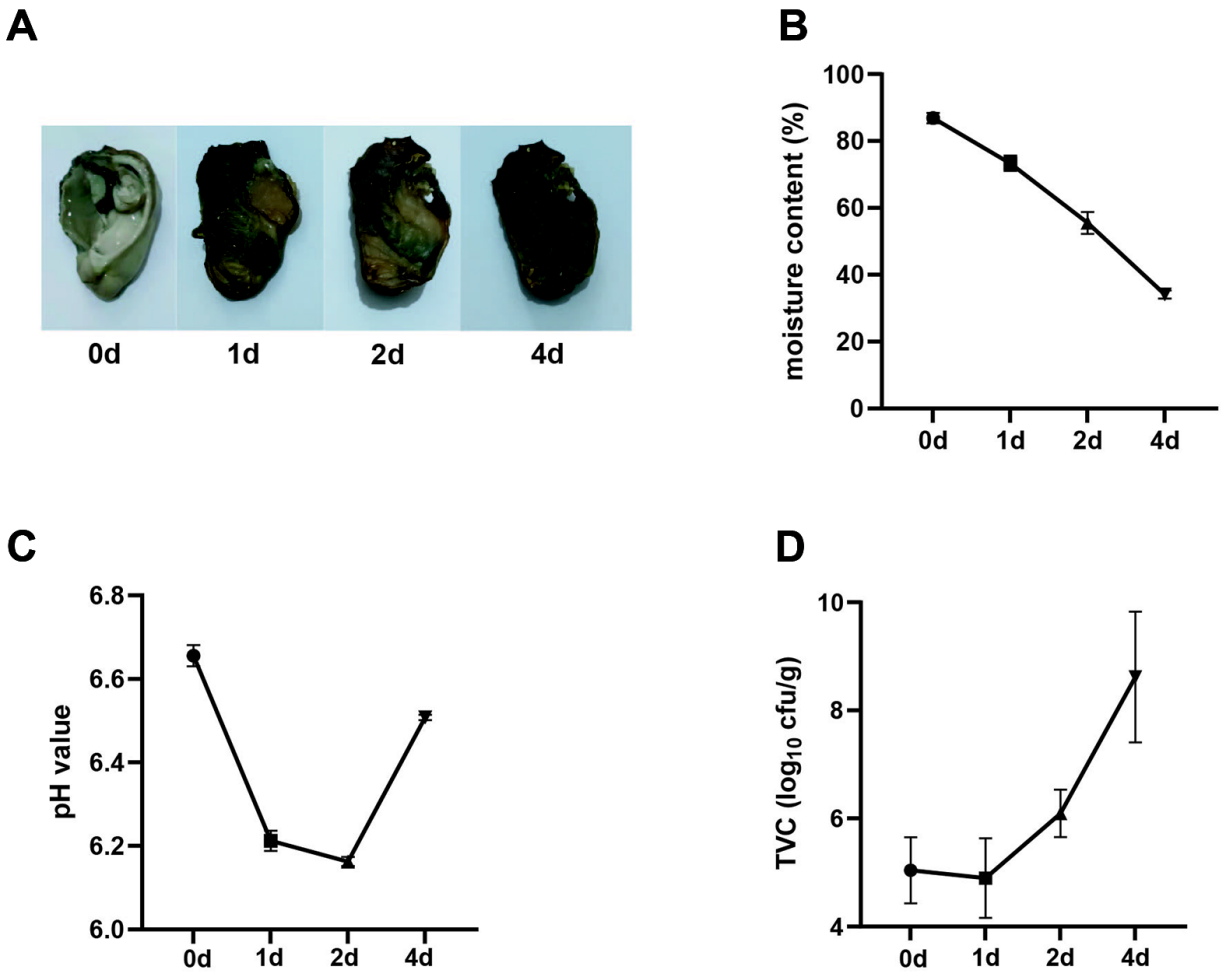
The changes in visual appearance, moisture content, pH levels, and TVC (Total Viable Count) of the samples during the sun exposure process. (**A**) Photographs of oysters at four time points: pre-sun exposure (OGT0, top left), 1 day after sun exposure (OGT1, top right), 2 days after sun exposure (OGT2, bottom left), and 4 days after sun exposure (OGT4, bottom right). (**B-D**) The changes in moisture content (**B**) pH levels (**C**) and TVC (**D**) of the oyster samples at the four time points mentioned above.

During sun drying, oysters undergo gradual desiccation, resulting in surface contraction, concomitant with progressive darkening of coloration, transitioning from an initial white hue to yellow at OGT1 and ultimately culminating in darkened pigmentation at OGT2 and OGT4 (Fig. 1A). The mosture content gradually decreased during preparation (Fig. 1B). The pH levels significantly decreased after sun exposure, with OGT1 and OGT2 exhibiting the lowest values, whereas a subsequent recovery was observed in OGT4 (Fig. 1C). Concurrently, the TVC showed a notable increase in the OGT4 group (Fig. 1D and Table S1). These results indicate that changes in the bacterial community or abundance require further investigation.

### Diversity of Bacterial Community in Dried Oysters

The 16s rRNA sequencing generated approximately 64.7 to 98.1 thousand paired-end reads per sample (Table S2). After quality control, the concatenation of paired-end reads into tags, and the removal of chimeric tags, approximately 61.1–94.3 effective tags per sample were obtained (Table S2). Among these, an average of 409 OTUs per sample were identified based on an average of 74.6% of taxon tags per sample (Table S2). The species accumulation curve nearly reached saturation (Fig. S1).

**Fig. 2:**
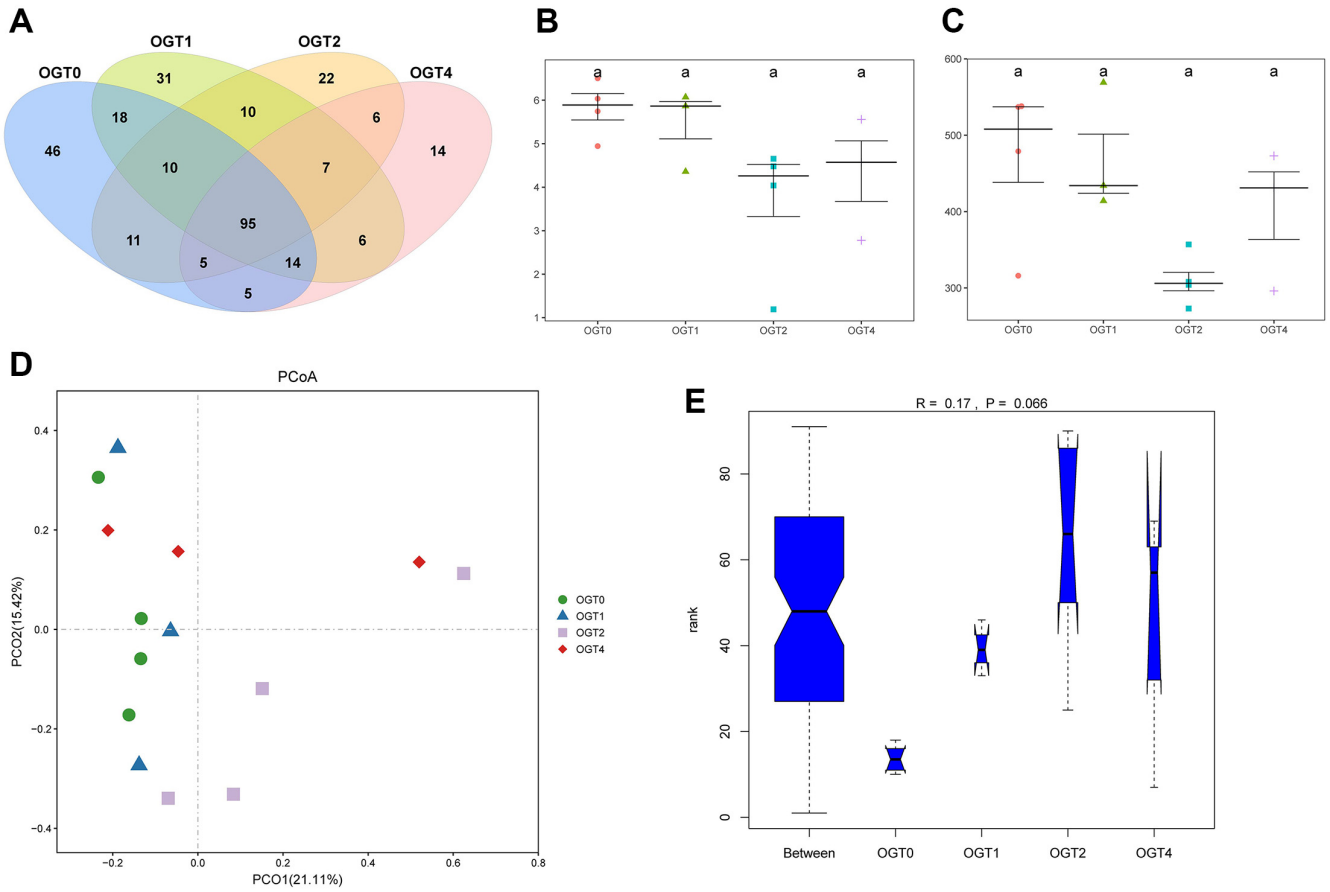
Global OTU and diversity analysis. (**A**) Venn diagram of all the time points at the OTU level. (**B-C**) Alpha diversity for Shannon index (**B**) and observed OTU (**C**). Different letters indicate statistical significance (*P* < 0,05), and same letters indicate no significance. (**D**) PCoA score plot based on binary Jaccard distances at genus level. Different point color and shapes represent samples at different time points. (**E**) The ANOSIM test based on binary Jaccard distances at genus level.

The intersection of OTUs revealed that there was a total of 95 shared OTUs across the four time points, with relatively fewer unique OTUs in each group (Fig. 2A). In addition, alpha diversity metrics, including the Shannon index (Fig. 2B) and observed species (Fig. 2C), did not exhibit significant differences among the four time points. PCoA indicated that samples from each time point displayed good repeatability with no distinct outlier samples, although the intergroup distances were relatively small (Fig. 2D). Furthermore, analysis of similarities (ANOSIM) suggested that intergroup differences were lower than intragroup differences (Fig. 2E). A previous study has shown that there are lower inter-individual and intra-group variability in the microbiome between Louisiana and Maryland oyster in America, which was consistent with our result [34].

### Taxonomic Shifts in Microbial Community during Preparation

**Fig. 3:**
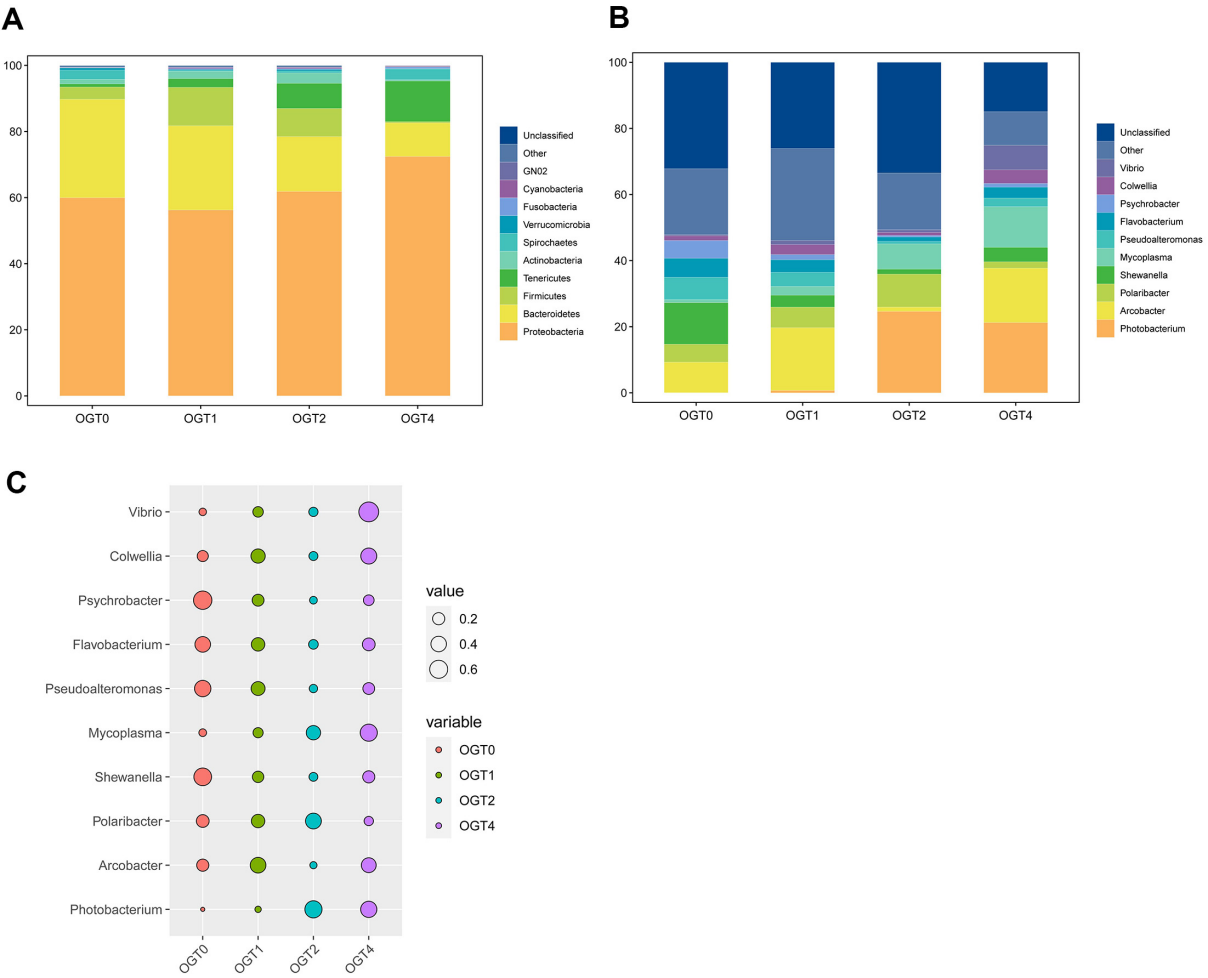
Microbial composition and indicator species analysis. (**A-B**) Average bacterial community composition of all samples at different time points at phylum (**A**) and genus (**B**) level, respectively. (**C**) Indicator species analysis at genus level. The size of the points was proportional to the relative abundance of the corresponding genus.

Taxonomic composition analysis revealed that the microbial community in the oyster samples during the sun-drying process was primarily composed of *Proteobacteria*, *Bacteroidetes*, *Firmicutes*, *Tenericutes*, and *Actinobacteria* at the phylum level (Fig. 3A).

Notably, *Proteobacteria* exhibited a relatively high abundance of OGT4, while *Bacteroidetes* and *Tenericutes* displayed gradually decreasing and increasing trends, respectively, across all four time points (Fig. 3A). A previous study demonstrated that *Proteobacteria* tended to be the most abundant microbial community and *Tenericutes* was also found in significant abundance in mangrove oysters Crassostrea gasar, which was consistent with our result [35]. Another study has shown that Bacteroidetes were mainly observed in the oyster *Pteria penguin* [36]. At the genus level, the predominant microbial genera included *Photobacterium*, *Arcobacter*, *Polaribacter*, *Shewanella*, *Mycoplasma*, *Pseudoalteromonas*, *Flavobacterium*, *Psychrobacter*, *Colwellia*, and *Vibrio*. Notably, certain bacteria within the genera *Shewanella*, *Arcobacter*, *Mycoplasma*, and *Vibrio* are potential human pathogens, with the latter three showing high abundances, particularly for OGT4 (Fig. 3B). Previous studies have shown that *Shewanella* *Arcobacter* and *Vibrio* were the most human pathogens isolated from oyster and exhibited great threaten toward human health [5, 37].

Indicator species analysis further highlighted that *Psychrobacter*, *Flavobacterium*, *Pseudoalteromonas*, and *Shewanella* were significantly more abundant in the oyster samples before sun-drying than that at other time points (Fig. 3C). Conversely, *Vibrio* and *Mycoplasma* genera exhibited significantly higher abundances after sun-drying than that at the other time points (Fig. 3C). Additionally, *Colwellia* and *Arcobacter* genera showed higher abundance at OGT1 and OGT4 compared to the others, *Photobacterium* displayed higher abundance at OGT2 and OGT4, while *Polaribacter* exhibited lower abundance at OGT4 than that at the other time points (Fig. 3C). A previous study showed that *Colwellia* exhibited a higher abundance in the oyster tissues other than gills during cold storage [38]. Another study has proved that *Arcobacter* was a common pathogen among oyster products [39]. Also, *Polaribacter* exhibited different abundances among various ways of diets in oyster [40].

### Metabolomic Profiling and Differential Analysis

**Fig. 4:**
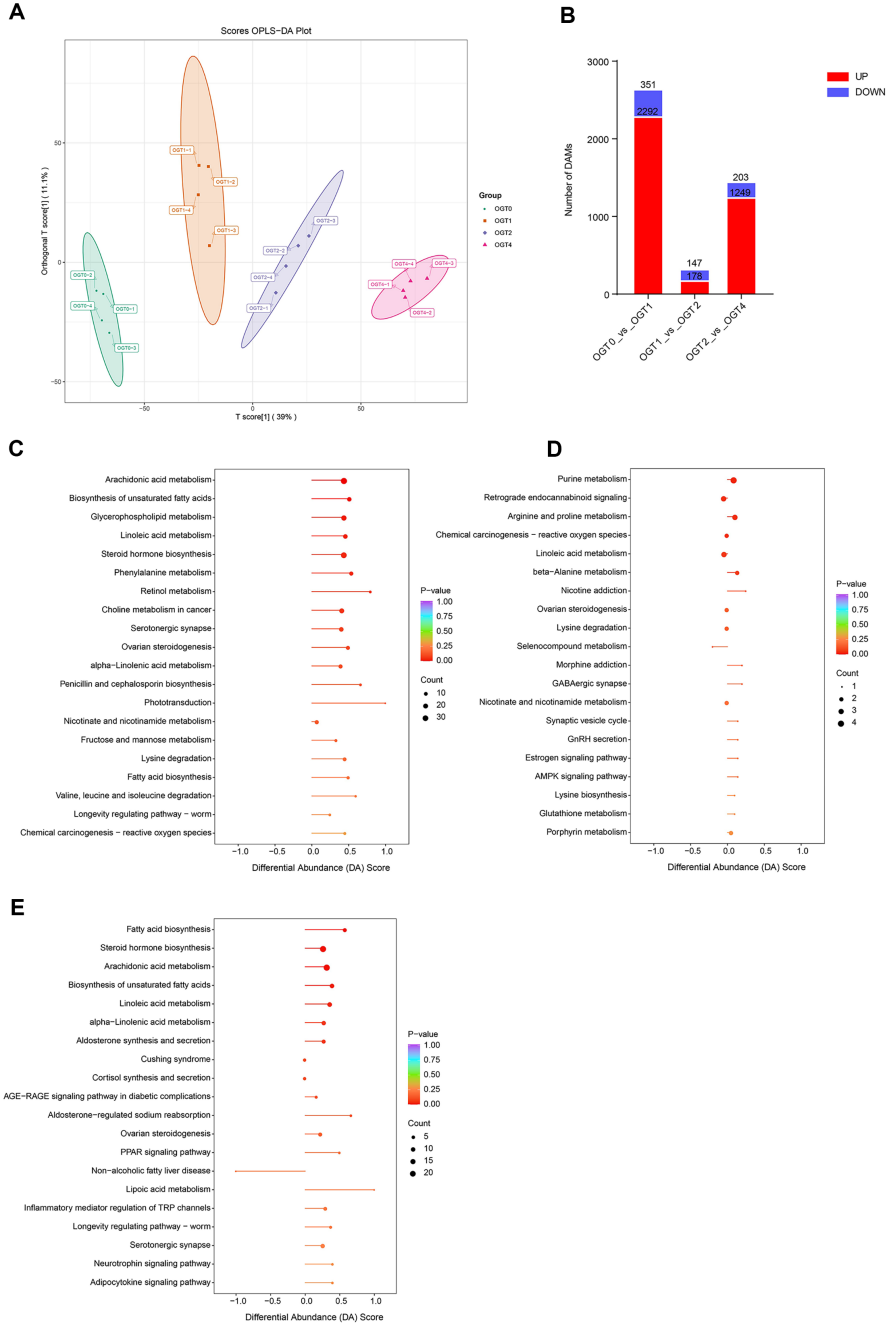
Metabolomic profiling of oyster samples during sun drying. (**A**) OPLS-DA score plot. (**B**) The bar chart depicting the number of differentially accumulated metabolites (**DAM**) between adjacent time points. (**C-E**) DA Score analysis of KEGG pathway between adjacent time points, including OGT0 vs. OGT1 (**C**), OGT1 vs. OGT2 (**D**), and OGT2 vs. OGT4 (E).

In oyster samples, 13,713 metabolites were identified, of which 7,571 were categorized as secondary metabolites (Table S3). Principal component analysis (PCA) indicated that intra-group samples clustered closely, exhibiting a gradual progression along the x-axis during sun-drying process (Fig. S2). OPLS-DA effectively discriminated between samples from different time points, where samples within the same time group clustered closely together, indicating significant intra-group homogeneity (Fig. 4A). Further identification of DAMs between adjacent time points revealed the most significant differences in the OGT0 vs. OGT1 comparison, with 2,643 DAMs (2,292 upregulated and 351 downregulated). In the OGT1 vs. OGT2 and OGT2 vs. OGT4 comparisons, 325 (178 upregulated and 147 downregulated) and 1,452 (1,249 upregulated and 203 downregulated) DAMs, respectively, were identified (Fig. 4B).

Furthermore, we performed a KEGG pathway-based differential abundance (DA) score analysis between adjacent time points, which indicated that compared to OGT0, OGT1 exhibited a significant increase in arachidonic acid metabolism, biosynthesis of unsaturated fatty acids, glycerophospholipid metabolism, linoleic acid metabolism, steroid hormone biosynthesis, phenylalanine metabolism, and retinol metabolism (Fig. 4C). The arginine and proline metabolism pathways were significantly elevated in the OGT2 group compared to those in the OGT1 group (Fig. 4D). A previous study has shown that the content of arginine was significantly increased during the drying process in oyster [41]. Similarly, compared to OGT2, the OGT4 group exhibited a significant increase in pathways related to steroid hormone biosynthesis, arachidonic acid metabolism, biosynthesis of unsaturated fatty acids, and linoleic acid metabolism (Fig. 4E). Previous studies have shown that arachidonic acid metabolism, biosynthesis of unsaturated fatty acids, and linoleic acid metabolism were remarkably higher than that in the fresh oyster group during drying process [42, 43].

**Fig. 5:**
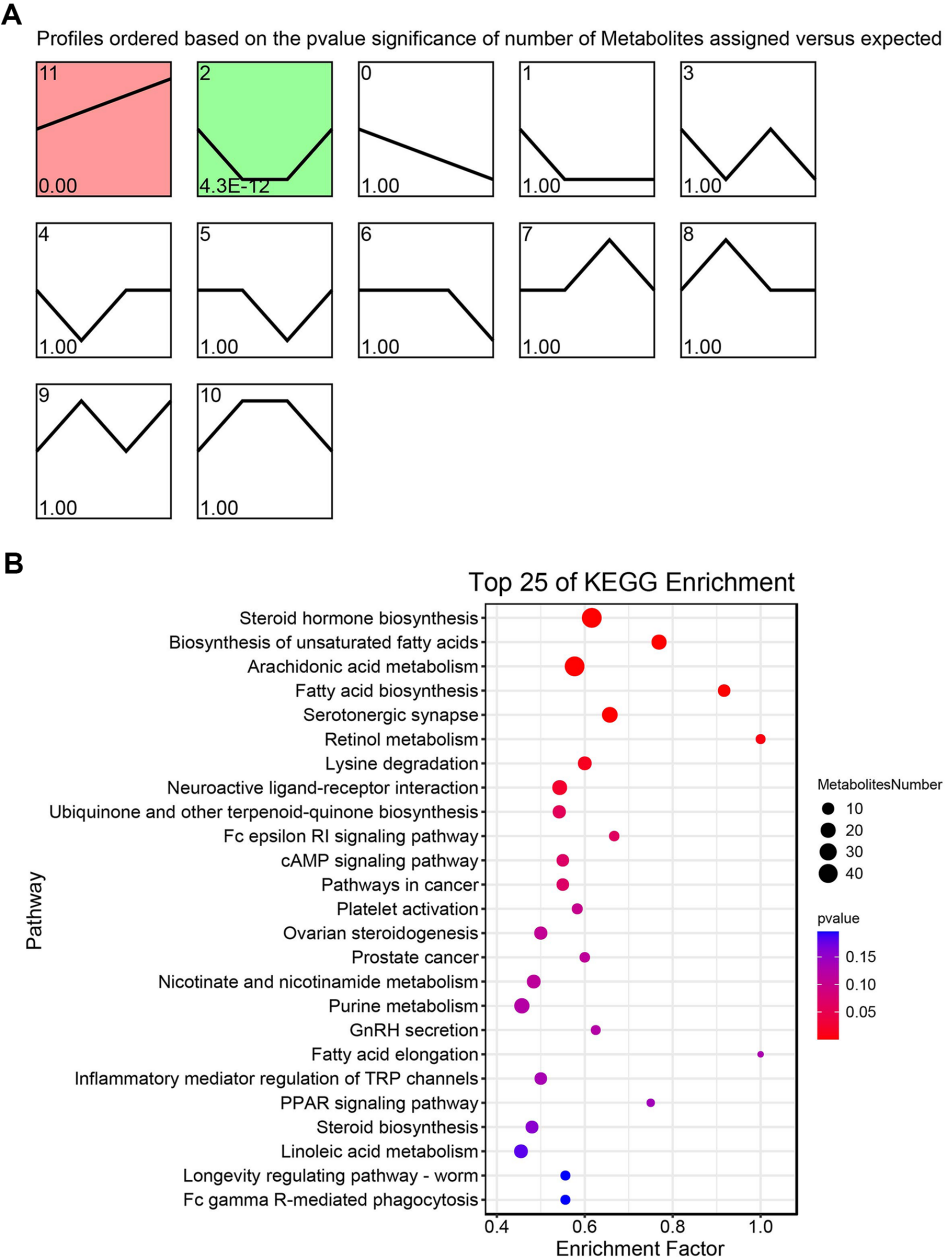
Identification of temporal metabolomic profiles. (**A**) The profiles of DAM identified by STEM. Significant profiles were highlighted with a colored background. (**B**) KEGG enrichment analysis of DAM in the above two significant profiles.

Temporal analysis of DAMs was conducted using STEM, revealing two significant temporal expression profiles, characterized by a gradual increase and downregulation, followed by a recovery trend (Fig. 5A). The DAMs in these two distinct temporal profiles were primarily enriched in pathways, such as steroid hormone biosynthesis, unsaturated fatty acid biosynthesis, arachidonic acid metabolism, fatty acid biosynthesis, and serotonergic synapses (Fig. 5B). A study has shown that the content of unsaturated fatty acid was significantly increased during the drying process of oyster [44]. Another study has shown that drying process led to a diminution in the proportionate concentration of fatty acids and concomitantly elevated the proportional concentration of glycerol phospholipids in *Crassostrea hongkongensis* fresh and dried products [45].

### Microbiome–Metabolome Interactions in Dried Oysters

**Fig. 6:**
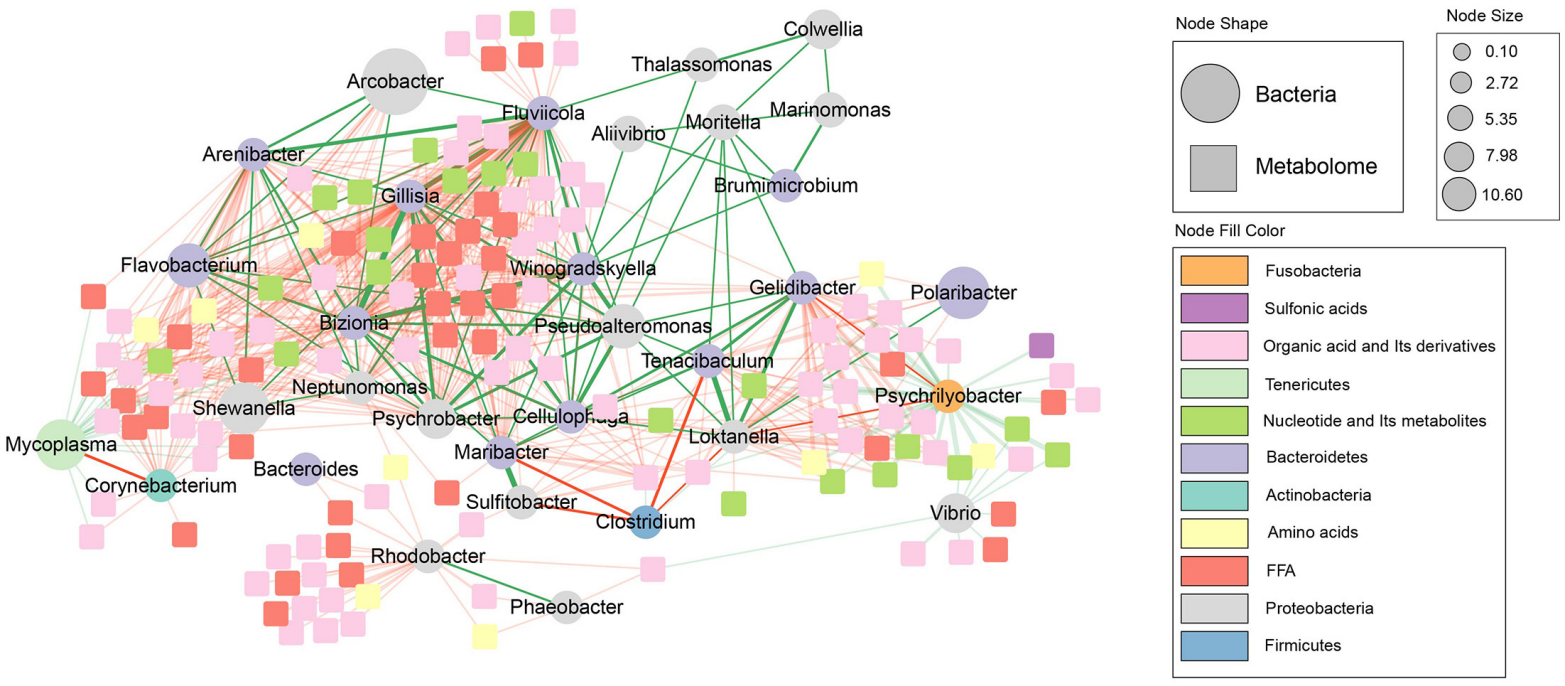
Construction of microbiome-metabolome interaction network. The circular and square nodes correspond to bacteria and metabolites, respectively, with node size being proportional to the abundance of the corresponding bacteria or metabolites. Nodes of different colors represent various metabolite categories or bacterial phyla. The edges are colored in red and green, indicating positive and negative correlations, respectively, with the thickness of the lines being proportional to the magnitude of the correlation coefficient.

To explore the interactions between the core microbiota and their associations with DAMs during the sun-drying process, a microbiome–metabolome correlation network was constructed at the genus level for all oyster samples (Fig. 6). The results revealed that highly abundant genera, such as *Shewanella* and *Arcobacter*, along with most other bacteria, exhibited a pattern of co-occurrence, whereas Clostridium and *Psychrilyobacter*, as well as *Mycoplasma* and *Corynebacterium*, were predominantly associated with exclusionary relationships (Fig. 6). Additionally, the genera *Mycoplasma*, *Psychrilyobacter*, and *Vibrio* showed negative correlations with a substantial number of metabolites in categories, including organic acids and their derivatives, nucleotides and their metabolites, free fatty acids (FFAs), and amino acids, whereas *Shewanella* and *Arcobacter* were positively correlated with these metabolite categories (Fig. 6). Previous studies have shown that *Mycoplasma*, *Psychrilyobacter* are negatively associated with organic acid and free fatty acids metabolism [46, 47]. Another study has shown that *Shewanella* and *Arcobacter* exhibited positive correlations with organic acid and free fatty acids metabolism [48-50]. Despite the limitations of these findings, and recognizing that the sample size of this study will limit our ability to find more meaningful results, more data are needed to explore the further interactions between microbiomes and metabolites in dried oysters.

## Conclusion

This investigation aims to examine the impact of the production process on the microbiota and metabolites in dried oysters. Employing 16s rRNA amplicon sequencing, the identification of 409 operational taxonomic units (OTUs) was conducted, revealing *Proteobacteria*, *Bacteroidetes*, *Firmicutes*, *Tenericutes*, and *Actinobacteria* as the predominant pathogenic bacteria in oyster samples. Analysis of the metabolomes in dried shrimp samples using LC-MS disclosed a noticeable increase in compounds associated with steroid hormone biosynthesis, arachidonic acid metabolism, biosynthesis of unsaturated fatty acids, and linoleic acid metabolism throughout the entire drying process. Subsequent examination of the relationship between metabolites and bacterial communities highlighted the prevailing coexistence of *Mycoplasma*, *Psychrilyobacter*, and *Vibrio*, demonstrating negative correlations with a significant number of metabolites across various categories, including organic acid and its derivatives, nucleotide and its metabolites, free fatty acids, and amino acids. Conversely, *Shewanella* and *Arcobacter* exhibited positive correlations with these metabolite categories. This comprehensive investigation provides valuable insights into the dynamic alterations within the microbiota and metabolites of dried oysters across diverse drying periods. These findings are expected to make a significant contribution to the advancement of production techniques and the formulation of enhanced safety measures for the processing of dried oysters.

## Supplemental Materials

Supplementary data for this paper are available on-line only at http://jmb.or.kr.
