# Supplementary material for: Dynamic Changes in Microbiome and Metabolome during Sun-Drying of Oysters (Crassostrea gigas), a Traditional Procedure in South China
Source: J Microbiol Biotechnol. 2024 Jul 17;34(12):2555–64. doi: 10.4014/jmb.2312.12033 (PMC11729336; doi:10.4014/jmb.2312.12033)
Supplement: Supplementary file 1 [file jmb-34-12-2555-supple.pdf]

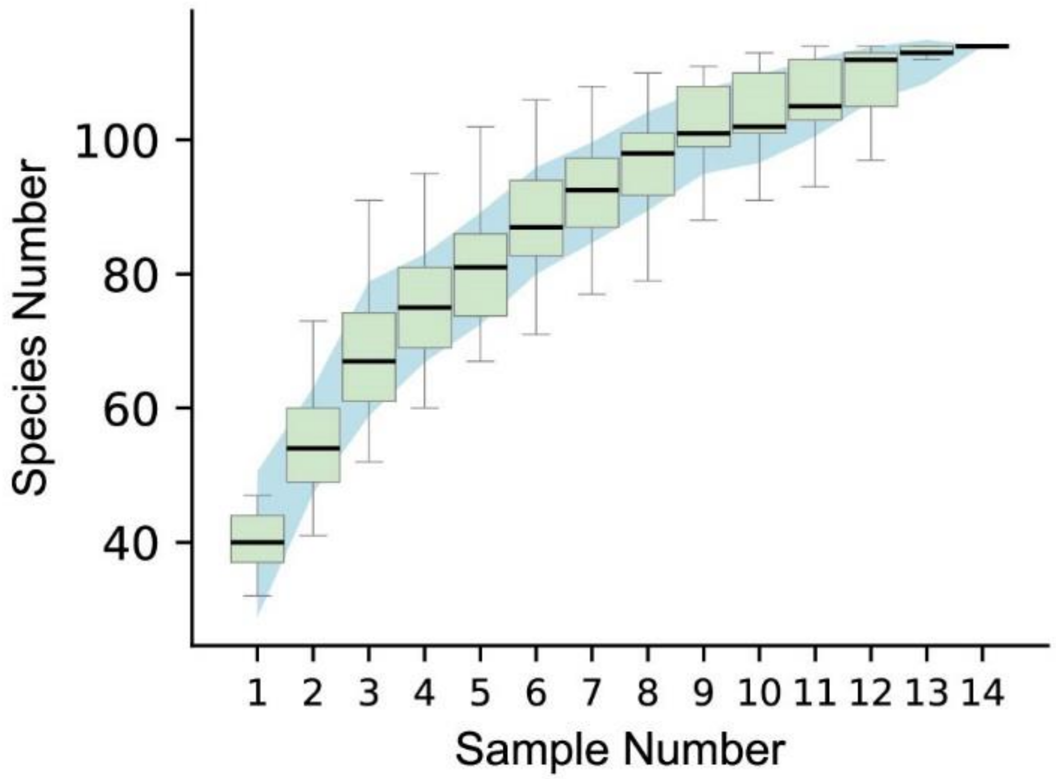


Figure S1. Species accumulation curve of all the oyster samples.

A

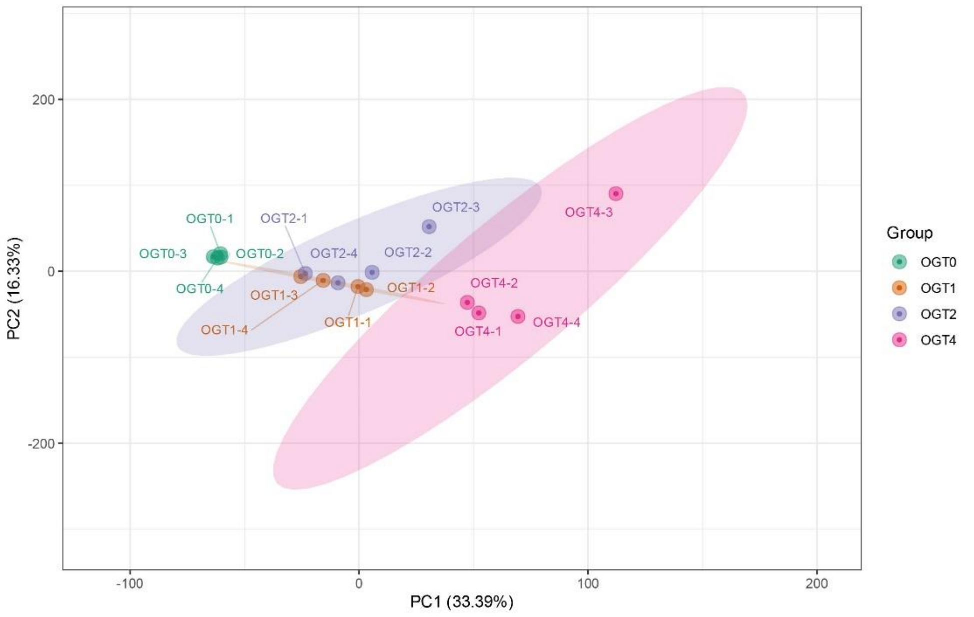


B

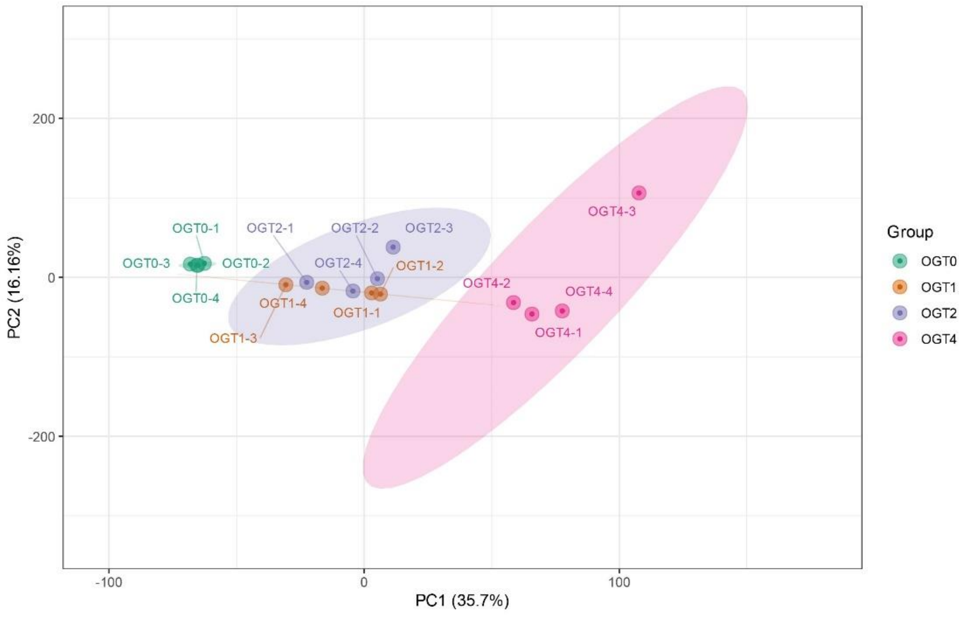


Figure S2. The scatter plot of PCA analysis for metabolomics data in positive (A) and negative (B) mode.

Table S1. Variation in TVC during dried osyters preparation.

| Sample Group | Replicate 1 | Replicate 2 | Replicate 3 | Replicate 4 |
|---|---|---|---|---|
| OGT0 | 3.78 | 3.60 | 3.70 | 4.00 |
| OGT1 | 4.41 | 2.60 | 4.08 | 3.70 |
| OGT2 | 4.46 | 3.85 | 3.95 | 3.30 |
| OGT4 | 5.53 | 5.54 | 5.64 | 5.74 |

Table S2. Statistics of 16S rDNA amplicon sequencing data.

| Sample Name | Raw PE | Clean PE | Raw Tags | Clean Tags | Chimera | Effective Tags | Effective Ratio (%) | Total Tags | Taxon Tags | Unclassified Tags | Singleton Tags | OTUs |
|---|---|---|---|---|---|---|---|---|---|---|---|---|
| OGT0-1 | 86786 | 86080 | 85120 | 84379 | 739 | 83640 | 96.37 | 83640 | 80271 | 0 | 3369 | 316 |
| OGT0-2 | 85540 | 84898 | 84045 | 83321 | 597 | 82724 | 96.71 | 82724 | 80517 | 0 | 2207 | 537 |
| OGT0-3 | 82957 | 82311 | 81150 | 79616 | 1094 | 78522 | 94.65 | 78522 | 74314 | 0 | 4208 | 538 |
| OGT0-4 | 81460 | 80860 | 79538 | 78258 | 1027 | 77231 | 94.81 | 77231 | 73390 | 0 | 3841 | 479 |
| OGT1-2 | 68255 | 67807 | 66461 | 64944 | 1414 | 63530 | 93.08 | 63530 | 57452 | 0 | 6078 | 414 |
| OGT1-3 | 98096 | 97368 | 96323 | 95097 | 757 | 94340 | 96.17 | 94340 | 91038 | 0 | 3302 | 434 |
| OGT1-4 | 77763 | 77195 | 75779 | 74647 | 562 | 74085 | 95.27 | 74085 | 71462 | 0 | 2623 | 569 |
| OGT2-1 | 82586 | 82013 | 80931 | 80150 | 150 | 80000 | 96.87 | 80000 | 78528 | 0 | 1472 | 308 |
| OGT2-2 | 74032 | 73474 | 72485 | 71507 | 260 | 71247 | 96.24 | 71247 | 69324 | 0 | 1923 | 273 |
| OGT2-3 | 85777 | 85091 | 83987 | 83044 | 428 | 82616 | 96.31 | 82616 | 80915 | 0 | 1701 | 304 |
| OGT2-4 | 80504 | 79870 | 79049 | 78459 | 612 | 77847 | 96.7 | 77847 | 74670 | 0 | 3177 | 357 |
| OGT4-1 | 64712 | 64156 | 63056 | 61585 | 447 | 61138 | 94.48 | 61138 | 55460 | 0 | 5678 | 431 |
| OGT4-2 | 91893 | 91188 | 90451 | 89889 | 4122 | 85767 | 93.33 | 85767 | 77737 | 0 | 8030 | 296 |
| OGT4-4 | 85578 | 84823 | 83557 | 82464 | 789 | 81675 | 95.44 | 81675 | 79282 | 0 | 2393 | 473 |

Table S3. The statistics of identified metabolites.

| Ion mode | All | C18_positive | C18_negative |
| --- | --- | --- | --- |
| Number of metabolites | 9,321 | 4,627 | 4,694 |
| Number of metabolites identified at the secondary level | 6,301 | 3,318 | 2,983 |
